# Emotional and Social Outcomes of the Teaching Personal and Social Responsibility Model in Physical Education: A Systematic Review and Meta-Analysis

**DOI:** 10.3390/children11040459

**Published:** 2024-04-11

**Authors:** Yalin Aygun, Hulusi Boke, Fatma Hilal Yagin, Sakir Tufekci, Talha Murathan, Ertugrul Gencay, Pablo Prieto-González, Luca Paolo Ardigò

**Affiliations:** 1 Department of Sport Management, Faculty of Sport Sciences, Inonu University, 44280 Malatya, Turkey; yalin.aygun@inonu.edu.tr (Y.A.); sakir.tufekci@inonu.edu.tr (S.T.); talha.murathan@inonu.edu.tr (T.M.); 2 Yasar Oncan Secondary School, Ministry of National Education, 44900 Malatya, Turkey; 754785@meb.k12.tr; 3 Department of Biostatistics and Medical Informatics, Faculty of Medicine, Inonu University, 44280 Malatya, Turkey; hilal.yagin@inonu.edu.tr; 4 Department of Recreation Management, Faculty of Sport Sciences, Istanbul Yeni Yuzyilas University, 34010 İstanbul, Turkey; ertugrul.gencay@yeniyuzyil.edu.tr; 5 Sport Sciences and Diagnostics Research Group, GSD-HPE Department, Prince Sultan University, Riyadh 11586, Saudi Arabia; pprieto@psu.edu.sa; 6 Department of Teacher Education, NLA University College, 0166 Oslo, Norway

**Keywords:** meta-analysis, teaching personal and social responsibility, physical education, product outcomes

## Abstract

Context: In today’s ever-changing world, fostering personal and social responsibility is essential for building strong and compassionate communities. This study aimed to provide a quantitative synthesis focusing on the emotional and social outcomes of Teaching Personal and Social Responsibility (TPSR) model-based Physical Education (PE) programs. Methods: A comprehensive literature review covering the period from November 2022 to September 2023 identified 637 articles published between 2005 and 2023. Of these, 20 met the inclusion criteria. Data from these articles were coded, and a comprehensive meta-analysis was conducted, incorporating 28 effect sizes. Methodological quality was assessed using the Medical Education Research Study Quality Instrument. Hedge’s g served as the effect size measure and emotional and social outcomes subgroups were consolidated. Heterogeneity was evaluated with Cochran’s Q and I^2^. Meta-regression and ANOVA-like models addressed categorical moderators, whereas publication bias was assessed through funnel plot, failsafe number, and Egger’s linear regression. Results: A significant and positive effect of the TPSR model on product outcomes (Hedge’s g = 0.337, 95% CI = 0.199 to 0.476) was found. Despite considerable heterogeneity (I^2^ = 83.830), a random effects model was justified. Assessment of publication bias indicated a low likelihood. Moderator analyses revealed that publication countries significantly influenced the effect, with stronger effects in Turkey. Publication type (article vs. thesis) also played roles in moderation. The meta-regression analyses did not reveal significant effects for the grade level, duration of intervention, publication year or sample size on the TPSR model’s impact on product outcomes. The TPSR model positively impacts emotional and social outcomes in PE, enhancing children’ skills and behaviour. However, variations across cultures highlight the need for further research, considering limitations like language constraints and potential biases in study selection and data extraction.

## 1. Introduction

The Teaching Personal and Social Responsibility (TPSR) model was initially developed to leverage physical activity in order to promote the acquisition of meaningful, fundamental and transferable life skills, norms, values, character and responsibility, particularly among disadvantaged or underprivileged children [1]. The TPSR model is rooted in two unwavering convictions: “putting kids first” ([2], p. 36) and “helping kids become better people” ([1], p. 18). The general objective of the TPSR model is to cultivate the personal, social and emotional well-being of children (e.g., a systematic literature review by Shen et al. [3]), enhancing their capacity for excellence and strengths within a prospective approach [4,5,6,7]. Moreover, the TPSR model stands out in physical education (PE) by promoting children’ holistic development. It fosters personal and social skills, cultivates positive character traits, boosts self-efficacy and confidence, reduces behavioural problems, and prepares children for lifelong physical activity. This structured approach, emphasizing responsibility and personal growth, makes TPSR a valuable framework for educators to create impactful and meaningful PE experiences [3,6].

The TPSR model, implemented within the structured and intentional context of PE and other related programs, has consistently evolved through ongoing revision and refinement [1,8,9]. In 1985, Hellison [8] interiorised the term “Taking Personal and Social Responsibility” to accentuate the program’s avoidance of a rigid set of actions. Nevertheless, in 2003, he transitioned to the term “Responsibility Model,” a modification attributed to its pervasive acknowledgement among teachers, educators, and youth workers [9]. Eventually, the “Personal and Social Responsibility” approach [1] has gained broad acceptance, buttressed by numerous scholarly works, often resulting in assorted positive outcomes (e.g., behavioural, social, emotional and academic development; [3]) among children in in-school [10] and out-of-school [11] settings.

Featuring a flexible instructional structure, TPSR model-based programs are organised into five pivotal and hierarchical levels: (1) respect for the rights and feelings of others; (2) self-motivation; (3) self-direction; (4) caring and (5) transfer “outside the gym” [1]. The initial four tiers delineate the fundamental tenets of the PE programs grounded in the TPSR model, encompassing a robust stakeholder–student rapport, the empowerment of students, the fusion of accountability and the facilitation of the transference of accountability [9]. Hellison ([9], p. 34) articulated the fifth level (transfer) as “the application of the four other levels to outside the program—on the playground, at school and home and on the street”. He remarked it as “the original impetus for developing TPSR” ([1], p. 21). Understanding how to effectively and consistently facilitate the five levels of responsibilities is crucial to the success of TPSR model-based programs. Furthermore, the effectiveness of a TPSR model-based program, as per the logic model of Izzo et al. [12], relies on the progressive achievement of immediate, intermediate and ultimate program outcomes. For instance, highlighting social and emotional outcomes, acknowledged as vital components in TPSR model-based programs, designates them as intermediate outcomes [3]. Moreover, fostering program–family collectivism [13,14] and taking into account socio-cultural factors may influence TPSR programming and its transfer, especially within the context of mainstream school system support and cultural norms and values [15]. Additionally, establishing a physically and emotionally safe environment could prevent traumatic experiences and enhance children’s sense of belonging to the programs. This is crucial for the success of their process elements [16].

Except for a reduced number of studies (e.g., Richards et al. [17]) proposing the combination of the TPSR model with the skill themes approach, most TPSR studies have been grounded in the self-determination theory. This asserts that motivation serves as a pivotal mechanism in fulfilling and empowering specific psychological needs, namely competence, autonomy and relatedness [18,19], mainly addressed in National PE Standard 5 [9,20]. Competence encompasses the sense of mastery and efficacy. Autonomy entails engaging in behaviours with a complete sense of initiative, ownership and volition, whereas relatedness involves a profound sense of belonging with others in a meaningful manner [21,22]. Within the TPSR model-based programs, children can experience autonomy and responsibility in decision making [23]. These enriching experiences typically unfold within small, voluntary, out-of-school or community-based settings, where children, despite confronting challenges and self-doubt, actively engage and participate [1]. 

At the forefront, TPRS model-based programs emphasise both procedural elements and the resulting product outcomes to foster optimal child development [24]. Assessing these two dimensions assists stakeholders, including educators, program leaders, parents and youth workers, in acquiring a comprehensive understanding of ongoing experiences [4] and offers profound insights into specific process elements that contribute to the product outcomes [3,7]. 

In this regard, there have been notable systematic literature review critiques on the TPSR model-based programs and practices assessing process elements (e.g., program duration, contextual framework and extensive range of population and culture) in in-school settings [10], out-of-school settings [11] and the global geographical distribution and representation [25]. Whereas these descriptive evaluations offer valuable insights, it is noteworthy that only one systematic literature review has specifically addressed the product outcomes in TPSR model-based programs, revealing four emerging themes: positive behavioural changes, improved inter-personal skills, enhanced emotional processes and improved academic performance [3].

Whereas the existing systematic literature reviews contribute to comprehending TPSR model-based programs, they have yet to closely examine their process elements or product outcomes, opting for a meta-analysis approach. Furthermore, the TPSR model-based programs prioritise social and emotional development as crucial intermediate outcomes [3]. Based on this, the primary purpose of this study is to make a quantitative synthesis examining the emotional and social outcomes of programs based on the TPSR model in the field of PE. The secondary aim of the study is to identify potential moderators such as publication country, grade level, publication type, intervention duration (weeks), publication year, and sample size, to investigate how these contextual factors affect the effectiveness of TPSR programs. Examining and understanding these moderators is critical to explaining how program outcomes may vary across specific contexts. To this end, this study formulated two broad research questions: (1) what is the impact of TPSR model-based programs on emotional and social outcomes? and (2) what study characteristics moderate the effects outlined in research question 1?

## 2. Materials and Methods

### 2.1. Protocol 

A systematic review, including a meta-analysis, was run following the PRISMA (Preferred Reporting Items for Systematic Reviews and Meta-analysis) guideline [26].

### 2.2. Eligibility Criteria

The PICOS (Population, Intervention, Comparison, Outcomes, Study design) as a framework to formulate eligibility criteria was utilised. This approach ensured that our inclusion and exclusion criteria were well-defined and rigorous, allowing us to select publications that were most relevant to our research (see Table 1; [27]).

### 2.3. Information Sources

Studies included published articles and theses written in English and Turkish. The collected studies in Turkish were sourced from ULAKBILM (Turkish Academic Network and Information Centre) and YÖK (Turkish Higher Education Council) Doctoral and Master Theses Database. The collected studies in English were sourced from ERIC, Google Scholar, Grey Literature, Psych ARTICLES, Psych INFO, ProQuest Dissertations and Theses database, Sport DISCUS and Web of Science.

### 2.4. Search

The time period for conducting literature review spans from November 2022 to September 2023. The search keywords combination for the TPSR were twofold: responsibility model (personal and social responsibility, responsibility model, teaching personal and social responsibility, personal and social responsibility program) and physical education (school, in-school, physical education, primary/primary-secondary/secondary/high school). The initial search yielded a total of 721 articles.

### 2.5. Study Selection

**Figure 1:**
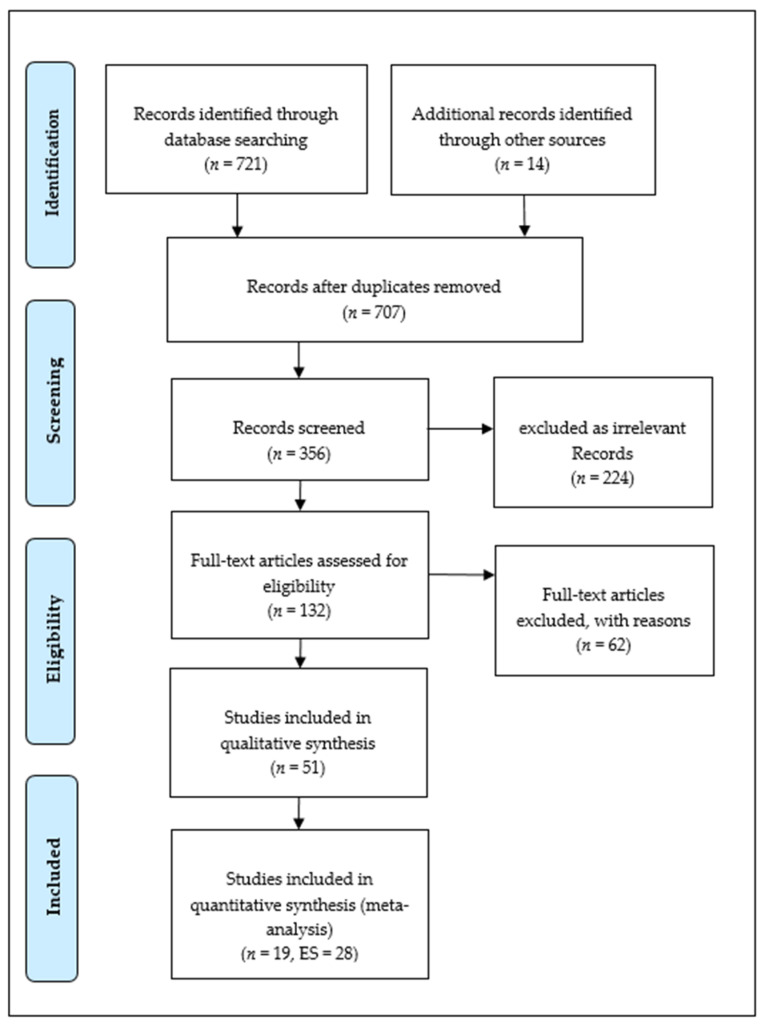
Study flow diagram utilizing PRISMA.

This systematic review and meta-analysis encompass studies that investigated the effect of TPSR model-based programs on students’ product outcomes, focusing on social and emotional processes. As the TPSR model was finalized in the early 2000s and subsequently gained widespread use, the database search period was restricted to the years 2005–2023. The data of the included publications were selected by the initial author and recorded in a standardised format. In cases of discrepancies in decisions regarding study selection, consultation with the second author was sought for resolution. After carefully examining the relevant literature’s title, abstract and full text, redundant publications lacking precise data were removed. Ultimately, 17 (1 in Turkish) articles and 2 Turkish theses meeting the specified criteria were selected, yielding 28 effect sizes (Figure 1).

Following the screening process, the collected publications underwent feature coding to capture various elements, including author (year), publication country (US, Serbia, Spain, Taiwan, Turkey), grade levels (encompassing primary school, secondary school, primary-secondary school and high school), publication type (article and thesis), duration of the TPSR model-based programs intervention measured in weeks, study year and sample size. Additional details can be seen in Table 2. The inter-coder reliability index for Cohen’s Kappa was computed as 0.88 [32,33,34], demonstrating substantial agreement. Additionally, the inter-coder reliability index based on the approach proposed by Miles and Huberman [35] was determined to be 0.96, indicating excellent agreement between the coders.

### 2.6. Data Collection Process

Regarding the data collection process from the selected studies, various parameters, including sample size, mean, standard deviation, t-value, *p*-value and additional pertinent metrics, were recorded both prior to and after the implementation of the TPSR model based programs (pre- and post-test). This information was obtained directly from the texts provided in the studies. The authors were contacted to request the related information in those studies where the data were unavailable. When the authors did not respond, descriptive data were obtained using the GetData Graph Digitizer (version 2.24) and Plot Digitizer programs (version 2.4). When the information was flagged or incomplete, it was not included in the meta-analysis.

### 2.7. Data Items

The variables analysed in the meta-analysis of the present study were emotional and social outcomes resulting from TPSR model-based programs. Combining two or more reported subgroups into a unified group can be beneficial in certain situations. This is particularly necessary when a study presents separate sample sizes, means and standard deviations for distinct subgroups, such as men and women. Pooling this information facilitates the calculation of a single sample size, mean and standard deviation for each intervention group. We applied the Cochrane data combination formula [56] to amalgamate data from all included studies. Subsequently, scores for emotional outcomes (e.g., self-esteem, goal setting, decision-making, sense of trust, understanding of fair play assistance, loyalty, awareness of empathy) and social outcomes (e.g., meeting people, making friends, communication, prosocial behaviour, respect and competence), as defined by the TPSR model, were aggregated into subgroups.

### 2.8. Study Risk of Bias Assessment

The publication bias assessment involved utilising a funnel plot, the failsafe number (Nfs) method by Khoury et al. [57] and linear regression, as outlined by Egger et al. [58]. A low risk of publication bias is suggested when the following conditions are met: (1) effect sizes are primarily within the funnel and exhibit symmetry along its axis, (2) the failsafe number (Nfs) is below 5k + 10 (where k represents the number of original studies), and (3) Egger’s linear regression intercept is both non-significant and proximate to zero.

### 2.9. Additional Analyses

The meta-analysis was conducted using the Comprehensive Meta-analysis (CMA 3.3) software developed by Borenstein et al. [59].

#### 2.9.1. Study Quality Assessment

The methodological quality of all included studies was assessed utilizing the widely recognized Medical Education Research Study Quality Instrument (MERSQI) [60]. The MERSQI assesses the methodological rigor of a study by examining 10 items categorized into six dimensions: study design, sampling, data collection, assessment instrument validity, data analyses and outcomes. In alignment with the PE context, the MERSQI tool underwent slight modifications, explicitly excluding the assessment of potential/healthcare outcomes. This adjustment resulted in a revised maximum score of 17, whereas the original scoring range for study quality typically spans from 5 to 18. The overall methodological quality score for each study is displayed in Table 2.

#### 2.9.2. Effect Size Computation

Hedge’s g served as the chosen effect size measure [61], computed based on sample sizes (n_C_, n_E_), means (M_C_, M_E_) and standard deviations (SD_C_, SD_E_) extracted from the pre- and post-tests of the experimental groups. The eligible studies (*n* = 12) in this systematic review included randomised controlled trials (RCT; *n* = 11) or semi-RCT designs (*n* = 17).

#### 2.9.3. Heterogeneity Test

The heterogeneity of effect sizes was assessed through Cochran’s Q statistic and corresponding *I*^2^ value. When *I*^2^ is equal to or greater than 75, significant heterogeneity is observed in the studies, indicating the suitability of a random effects model over a fixed effects model. This circumstance also underscores the need to evaluate potential moderators [62]. Disparities in populations and measures across studies could impact the effect size, further justifying the adoption of a random effects model and the assessment of potential moderators.

Moreover, models akin to ANOVA can be computed using a categorical moderator variable [63]. The emphasis lies in comparing group mean effect sizes for two categories (e.g., publication type) or three or more categories (e.g., grade and country). In the presence of significant heterogeneity, a commonly employed practice in meta-analysis is applying a meta-regression model to clarify the heterogeneity by including study-level covariates [64,65]. Within this framework, meta-regression analyses were conducted with three continuous variables: duration of intervention (weeks), publication year and sample size.

## 3. Results

### 3.1. Summary of Data Description

**Figure 2:**
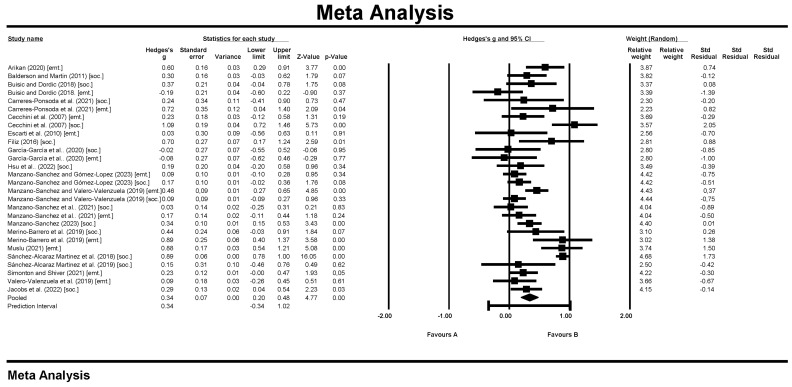
Forest plot for the random effects model [36,37,38,39,40,41,42,43,44,45,46,47,48,49,50,51,52,53,54,55,59].

The literature review identified 19 convenient studies encompassing 3040 students, with individual study participant ranges varying from 17 to 714. Within these studies, 28 independent effect sizes were extracted, as detailed in Table 2 and graphically represented in Figure 2.

### 3.2. Effect Size and the Homogeneity Test

The meta-analysis of the 28 independent effect sizes produced an overall Hedge’s g of 0.337 (95% CI = 0.199 to 0.476; z = 4.775, *p* < 0.000), revealing a significant and positive effect of the TPSR model on product outcomes. The Cochrane’s Q value of 166.981 was statistically significant (*p* < 0.000, *I*^2^ = 83.830), indicating considerable heterogeneity among effect sizes across the studies and advocating for the application of a random effects model.

### 3.3. Assessment of Publication Bias

**Figure 3:**
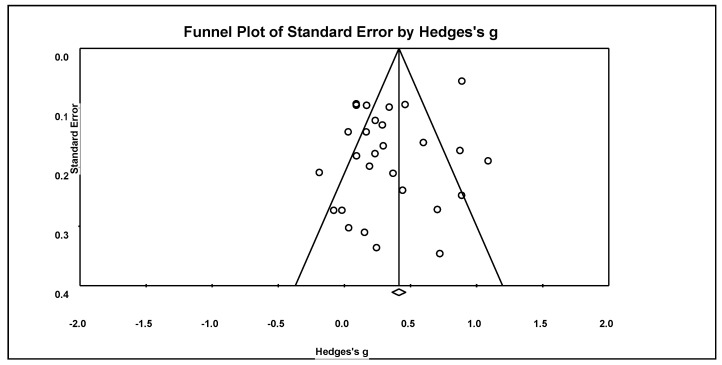
Funnel plot of effect sizes of the betas between TPSR model and product outcomes.

The collective analyses of the funnel plot, Nfs, Begg and Mazumdar rank correlation, and Egger’s regression consistently indicate a low likelihood of publication bias. Notably, the funnel plot revealed that the distribution of the 28 effects was predominantly within the expected range and symmetrically positioned around its axis, as illustrated in Figure 3. An Nfs value of 1060 significantly surpassed the failsafe criteria of 150 (calculated as (5 × 28) + 10). The rank correlation test reported a Kendall’s tau of 0.13 with a non-significant 2-tailed *p*-value of 0.31. Moreover, the Egger’s regression intercept registered at 1.71, with a non-significant two-tailed *p*-value of 0.09.

### 3.4. Moderator Analyses

Due to the significant heterogeneity observed in the effect sizes (see Table 3), we conducted tests for potential moderators, including product outcomes (social and emotional processes), publication country, grade, publication type, duration of intervention (week), publication year and sample size (outlined in Table 4).

#### 3.4.1. Publication Country 

Publication country moderated the effect of the TPSR model on product outcomes (*Q_BET_* = 14.603, *df* = 4, *p* < 0.05; see Table 4). This effect was stronger among publications in Turkey (*g* = 0.727, 95% CI = 0.515 … 0.939) than in Spain (*g* = 0.323, 95% CI = 0.140 … 0.506) Serbia (*g* = 0.091, 95% CI = −0.461 … 0.642), Taiwan (*g* = 0.193, 95% CI = −0.200 … 0.586) and USA (*g* = 0.269, 95% CI = 0.115 … 0.422).

#### 3.4.2. Grade

The effect sizes did not differ significantly among grade levels (*Q_BET_* = 0.728, *df* = 3, *p* > 0.05; see Table 4). Therefore, grade level did not moderate the effect of the TPSR model on product outcomes.

#### 3.4.3. Publication Type

Publication type (article vs. thesis) moderated the effect of the TPSR model on product outcomes (*Q_BET_* = 10.299, df = 1, *p* < 0.05; see Table 4). This effect was stronger among students in theses (*g* = 0.833, 95% CI = 0.545 … 1.121) than in articles (*g* = 0.307, 95% CI = 0.164 … 0.450).

#### 3.4.4. Duration of Intervention (Week)

Meta-regression of g onto duration of the intervention (week) did not show significant effects of the TPSR model on product outcomes (*Q_Model_* (1, k = 28) = 1.66, *p* > 0.05; see Table 5).

#### 3.4.5. Publication Year

Meta-regression of *g* onto publication year did not show significant effects of the TPSR model on product outcomes (*Q_Model_* (1, k = 28) = 1.21, *p* > 0.05; see Table 5).

#### 3.4.6. Sample Size

Meta-regression of g onto sample size did not show significant effects of the TPSR model on product outcomes (*Q_Mode_*_l_ (1, k = 28) = 2.79, *p* > 0.05; see Table 5).

## 4. Discussion

This meta-analysis and systematic review of 19 studies of 3040 participants and 28 effect sizes showed an overall TPSR model effect of 0.337 on emotional and social outcomes, which supports the efficacy of the TPSR model in the context of PE learning. Furthermore, publication country and publication type moderated the effect of the TPSR model on product outcomes, contributing to a comprehension of non-significant or even adverse effects.

### 4.1. The Teaching Personal and Social Responsibility Model and Product Outcomes

The TPSR model effect size of 0.337 on product outcomes is evident in the literature’s initial and ultimate stages of the meta-analysis result. This avant garde finding is consistent with the product outcomes of the TPSR model-based programs, which encompass social and emotional outcomes [3,10]. Shen et al. ([3], p. 101), aptly describe these outcomes as ‘labelled as intermediate outcomes’ of the TPSR model. Therefore, the TPSR model-based programs demonstrate notable efficacy in augmenting PE learning. This fosters the utilization and development of the TPSR model for teachers, educators, youth and youth workers, ultimately enhancing their effectiveness. Regardless, the preliminary effect size indicates that dedicating resources to the design and implementation of TPSR model-based programs could enhance students’ emotional and social outcomes.

TPSR model-based programs empower children to comprehend and manage their emotions through meaningful interactions with peers and adults [3,66,67]. This, in turn, assists them in advancing their abilities to recognise, express, regulate and navigate emotions, especially within the collaborative context of PA [3,10,68]. The instructional framework offered by TPSR model-based programs serves as a crucial catalyst in the development of relationships, giving rise to personalised feedback, criteria-based self-control, self-esteem, self-confidence [3], delayed gratification [40,69], and an elevated sense of empathy and concern for children [70]. In a 16-week study conducted in Turkey with 162 students, Arikan (2020) [36], reported a noteworthy impact of the TPSR model-based programs on the advancement of children’s emotional intelligence levels. In a qualitative research investigation utilising the TPSR model in a PE program with 36 high school students, Aksoy and Gürsel (2017) [71] identified an enhanced sense of trust, support and loyalty, fostering students’ heightened awareness of empathy and a deeper understanding of fair play within a framework of game-based physical activity. While Pope (2005) ([71], p. 273) acknowledges the challenge of observing and measuring the effect of TPSR model-based programs, noting that “it has come to mean so many things”, these studies provide compelling evidence that children involved in TPSR model-based programs demonstrated significant advancements in the critical emotional domain [3].

In addition to emotional outcomes, the social outcome in TPSR model-based programs can be designated as an intermediate outcome. The literature confirmed that the three levels of responsibilities (i.e., cooperation, assisting others and leadership) are intricately linked to social skills development [1]. TPSR model-based programs also create an environment for children to navigate and gain insights into their emotions through social interactions with peers and adults [3]. Previous studies focusing on personal and social responsibility in the classroom have proposed that improvements in indicators such as enjoyment and sportsmanship are attainable [40,69]. In Balderson and Sharpe’s study [72], conducted with fourth- and fifth-grade students from four elementary classes in an inner-city charter-school setting, it was reported that both the personal accountability and personal responsibility treatments were effective in the primary treatment setting, leading to positive changes in all managerial, off-task and positive social measures. Opstoel et al. [73], in a literature review, revealed that 54 studies discuss prosocial behavior, including aspects such as respect, empathy and sympathy, as outcomes of PA. In the same literature review, authors also found that interacting with others and forming meaningful relationships were articulated in 27 individual studies. Furthermore, Carreres-Ponsoda et al. [39] proposed that the TPSR model-based programs effectively improve personal and social responsibility and prosocial behaviour in youth soccer players compared to conventional sports teaching methods.

### 4.2. Publication Type and Country

Publication type (article vs. thesis) and publication country (Turkish) played roles in moderation. The effect size of TPSR on emotional and social outcomes was much larger for students in the Turkish thesis than for students in all individual articles, regardless of country. Gordon and Beaudoin [25], who investigated the global adoption of TPSR across 31 countries, noted a significant diversity in the methods, locations and target audiences for program delivery. They assert that implementing TPSR in various countries poses challenges in maintaining fidelity to the model. Addressing these challenges necessitates continuous research, high-quality professional development and the establishment of communities of practice. Cultural and contextual factors specific to Turkey might influence how the TPSR is applied and how it transfers to product outcomes. This could explain the larger effect size observed in Turkish theses. This result implies the need to prioritise an in-depth exploration of the TPSR model-based programs across different geographical locations.

## 5. Limitations and Future Research Directions

Despite adhering to the PRISMA guideline, utilizing the PICOS framework, MERSQI, and reliability tests for formulating eligibility criteria, subjective judgments may still be involved in deciding which studies to include or exclude. The inclusion criteria restricted articles and theses to those written exclusively in English and Turkish, potentially introducing language bias and excluding relevant studies published in other languages. This limitation may impact the comprehensiveness and generalizability of the findings. We recommend that future meta-analyses incorporate studies in languages beyond Turkish and English to provide a more comprehensive and holistic understanding of the TPSR model-based programs in PE. While we utilized various databases to source studies, there might still be a bias towards databases that are more accessible or familiar to us. This bias could result in the omission of studies from less well known or specialized databases, potentially leading to a skewed representation of the literature. Furthermore, while a second author was consulted to resolve discrepancies in study selection and data extraction, the criteria for resolving conflicts may not be fully transparent or standardized, potentially allowing for subjective judgment.

Given the limited geographical scope of TPSR model-based studies, further research in diverse countries is crucial. This would enable the exploration of other potentially influential country-level attributes, such as cultural values, economic systems and political structures. However, the need for more scientific rigor can pose a significant limitation when exploring products associated with the TPSR model. Based on the meta-analysis, two key product outcomes that facilitate the success of TPSR model-based programs were identified, i.e., emotional and social outcomes. Future meta-analysis studies can further distinguish the impact of the TPSR model-based programs on children’s other product outcomes, including behavioral changes, academic performance and physical and psychomotor development. Lastly, the intended focus of our meta-analysis did not center on the process outcomes of the TPSR model. The meta-analysis recommends future TPSR model-based meta-analysis focusing on examining process outcomes that contribute to the success of TPSR model-based programs, such as learning environment, leadership and transfer.

## 6. Conclusions

The TPSR model emerges as a promising framework for fostering positive emotional and social development in children through PE programs. The observed enhancements in emotional intelligence, social skills, self-esteem, empathy, and prosocial behavior highlight the model’s effectiveness in addressing a crucial aspect of holistic child development. Furthermore, the study underscores the potential influence of contextual factors on program outcomes. The variation based on publication country and type suggests that TPSR’s effectiveness might be contingent on cultural norms and program delivery methods. This necessitates further research exploring how cultural adaptation and tailoring of TPSR interventions can optimize their impact across diverse settings. Looking forward, this study emphasizes the need for broader dissemination and implementation of TPSR model-based programs. By incorporating TPSR into PE curriculums across various settings, researchers can gain a deeper understanding of how the model’s impact varies based on specific contexts. Additionally, investigating both product outcomes (e.g., emotional and social changes) and process outcomes (e.g., intervention fidelity, teacher training effectiveness) will provide a more comprehensive picture of TPSR’s overall efficacy. In conclusion, the TPSR model presents a valuable tool for educators and program developers seeking to enhance the emotional and social well-being of children through PE. By acknowledging the influence of context and addressing the limitations of this study, future research can pave the way for optimized TPSR interventions that effectively promote holistic child development in diverse educational settings. 

## 7. Practical Applications

Educational institutions and youth organizations can implement TPSR model-based programs to enhance children’ emotional and social development within PE curricula. These programs provide a structured framework for promoting personal and social responsibility, fostering positive relationships, and cultivating important life skills among children. Professional development opportunities focusing on TPSR implementation can be offered to PE teachers and youth workers, emphasizing core principles and effective integration strategies. Cultural adaptation is essential when implementing TPSR model-based programs in diverse settings to ensure relevance and impact. Continued research and evaluation are needed to understand the effects of TPSR programs on various outcomes, while community collaboration strengthens implementation and sustainability. Advocacy for policy support can enhance the systemic impact of TPSR model-based initiatives, promoting personal and social responsibility among children and youth through PE and related efforts.

## Figures and Tables

**Table 1:** PICOS strategy for the inclusion and exclusion criteria.

Category	Inclusion Criteria	Exclusion Criteria
Population	School-aged children and youth of both sexes aged between 6 and 18.	Children under 6 and young adults or adults aged 19 and above (e.g., Scanlon et al., 2022 [28]).
Intervention	Product outcomes, particularly within the social and emotional domains, TPSR model-based programs.	Product outcomes, encompassing behavioural changes, academic performance and physical and psychomotor domains within TPSR model-based programs. Intervention impact and results relevant to the analyses of process outcomes within TPSR model-based programs. (e.g., Javier Sánchez-Alcaraz Martínez et al., 2014 [29]).
Comparison	Studies involving a minimum of two groups, consisting of either one experimental and one control group or two experimental groups.	Absence of a minimum of two groups, either one experimental and one control group or two experimental groups (e.g., Martinek and Lee, 2012 [30]).
Outcomes	Studies that reported sufficient statistical information required for conducting calculations, including essential parameters such as sample size, mean, standard deviation, t-value, *p*-value for the experimental and control groups.	Studies that did not report sufficient product outcome parameters and for which it was not possible to obtain such data after contacting their authors (e.g., Beale, 2016 [31]).
Study design	Randomized and non-randomized controlled studies.	Cross-sectional studies. Interventions published in sources classified as grey literature, such as reports, conference proceedings not subjected to peer review or publications not issued by commercial publishers (e.g., Hellison, 2011 [1]).

**Table 2:** Characteristics of the 19 studies included in the meta-analysis.

Author	Hedges’s g	Publication Country ^a^	Grade ^b^	Publication Type ^c^	Study Design ^d^	Duration of Intervention (Week)	Study Year	Sample Size	*Q*
Arıkan (2020) * [36]	60	4	1	1	1	16	2020	82	14
Balderson and Martin (2011) ** [37]	30	5	4	1	2	2	2011	74	10
Buišić and Đorđić (2018) * [38]	0.37	1	2	1	1	8	2018	45	11.5
Buišić and Đorđić (2018) ** [38]	−0.19	1	2	1	1	8	2018	45	11.5
Carreres-Ponsoda. et al. (2021) * [39]	0.24	2	1	1	1	28	2022	17	11.5
Carreres-Ponsoda. et al. (2021) ** [39]	0.72	2	1	1	1	28	2022	17	11.5
Cecchini et al. (2007) * [40]	0.23	2	4	1	1	8	2007	63	13.5
Cecchini et al. (2007) ** [40]	1.09	2	4	1	1	8	2007	63	13.5
Escarti et al. (2010) * [41]	0.03	2	2	1	2	34	2010	21	11.5
Filiz (2016) ** [42]	0.70	4	1	2	2	8	2016	28	11
García-García et al. (2020) * [43]	−0.02	2	2	1	2	20	2020	26	12
García-García et al. (2020) ** [43]	−0.08	2	2	1	2	20	2020	26	12
Hsu et al. (2022) ** [44]	0.19	3	4	1	2	18	2022	50	12.5
Jacobs et al. (2022) ** [45]	0.09	5	4	1	2	4	2019	120	11
Manzano-Sánchez and Gómez-López (2023) * [46]	0.17	2	3	1	2	20	2023	216	13
Manzano-Sánchez and Gómez-López (2023) ** [46]	0.46	2	3	1	2	20	2023	216	13
Manzano-Sánchez and Valero-Valenzuela (2019) * [47]	0.09	2	3	1	2	28	2019	227	13
Manzano-Sánchez and Valero-Valenzuela (2019) ** [47]	0.03	2	3	1	2	28	2019	227	13
Manzano-Sánchez et al. (2021) * [48]	0.17	2	4	1	2	32	2021	100	13
Manzano-Sánchez et al. (2021) ** [48]	0.34	2	4	1	2	32	2021	100	13
Manzano-Sánchez (2023) ** [49]	0.44	2	4	1	2	24	2023	205	12
Merino-Barrero et al. (2019) ** [50]	0.89	2	3	1	1	20	2019	35	12.5
Merino-Barrero et al. (2019) * [50]	0.88	2	3	1	1	20	2019	35	12.5
Muslu (2021) * [51]	0.89	4	2	2	2	12	2021	73	11.5
Sanchez-Alcaraz Martínez et al. (2018) ** [52]	0.15	2	3	1	1	12	2018	714	14
Sanchez-Alcaraz Martínez et al. (2019) ** [53]	0.23	2	3	1	1	16	2019	20	13
Simonton and Shiver (2021) * [54]	0.09	5	2	1	2	36	2021	135	15.5
Valero-Valenzuela (2019) * [55]	0.29	2	2	1	2	8	2019	60	15

^a^ I = Serbia, 2 = Spain, 3 = Taiwan, 4 = Turkey, 5 = USA; ^b^ I = high school, 2 = primary school, 3 = primary-secondary school, 4 = secondary school; ^c^ I = article, 2 = thesis; ^d^ I = RCT, 2 = semi-RCT. * = emotional outcomes. ** = social outcomes.

**Table 3:** Random-model of the effect of TPSR model on product outcomes.

			Homogeneity Test			Tau-Squared			Test of Null (Two Tailed)	
k	Mean g Effect size	95% CI for ES	Q(g)	*p*	I^2^	Tau^2^	SE	Tau	z	*p*
28	0.337	0.199 … 0.476	166.981	0.000	83.830	0.104	0.071	0.322	4.775	0.000

**Table 4:** TPSR model and product outcomes: univariate analyses of variance for moderator variables (categorical variables).

	Between-Group Effect (*Q_BET_*)	k	*N*	Mean *g* Effect Size	SE	95% CI for ES	Homogeneity Test within Each Group (QW)	*I* ^2^
Publication country	0.323 *							
Serbia		2		0.091	0.281	(−0.461, 0.642)	3.523	71.616
Spain		19		0.323	0.093	(0.140, 0.506)	143.541 ***	87.460
Taiwan		1		0.191	0.200	(−0.200, 0.586)	0.000	0.000
Turkey		3		0.727	0.108	(0.515, 0.939)	1.397	0.000
USA		3		0.269	0.078	(0.115, 0.422)	0.129	0.000
Grade	0.323 *							
High school		4		0.596	0.121	(0.360, 0.832)	1.360	0.000
Primary School		8		0.195	0.128	(−0.055, 0.445)	21.448 **	67.363
Primary-secondary school		8		0.397	0.151	(0.101, 0.692)	102.184 ***	93.150
Secondary school		8		0.318	0.095	(0.132, 0.504)	22.431 **	68.793
Publication Type	0.411 *							
Article		26		0.307	0.073	(0.164, 0.450)	157.939 ***	84.171
Thesis		2		0.833	0.147	(0.545, 1.121)	0.258	0.000

Index; *** = *p* < 0.001; ** = *p* < 0.01; * = *p* < 0.05.

**Table 5:** Univariate regression analyses of continuous variables (random effect model).

	Parameter	Estimate	SE	Z-Value	95% CI for *B*
Duration of intervention (week)	*β*_0_	−0.0088	0.0068	−1.29	(−0.0222, 0.0046)
	*β*_1_	0.5026	0.1424	3.53	(0.2234, 0.7818)
	Q*_Model_* (1, *k =* 28) = *p >* 0.05				
Publication year	*β*_0_	−0.0174	0.0158	−1.10	(−0.0484, 0.0136)
	*β*_1_	35.4744	31.9624	1.11	(−27.1708, 98.1195)
	Q*_Model_* (1, *k =* 28) = *p >* 0.05				
Sample size	*β*_0_	0.0006	0.0004	1.67	(−0.0001, 0.0013)
	*β*_1_	0.2565	0.0760	3.38	(0.1075, 0.4054)
	Q*_Model_* (1, *k =* 28) = *p >* 0.05				
